# Revisiting secondary prevention in coronary heart disease

**DOI:** 10.1016/j.ihj.2022.11.011

**Published:** 2022-11-29

**Authors:** Alben Sigamani, Rajeev Gupta

**Affiliations:** a Numen Health, Bengaluru, Karnataka, 560095, India; b Department of Preventive Cardiology and Medicine, Eternal Heart Care Centre & Research Institute, Jaipur, Rajasthan, 302017, India

**Keywords:** Coronary heart disease, Secondary prevention, Pharmacotherapy, Digital health

## Abstract

Secondary prevention in coronary heart disease is the prevention of occurrence of recurrent coronary events after clinical diagnosis. High level of adherence to secondary prevention interventions, especially aggressive lifestyle changes and pharmacotherapy can lead to significant decline in recurrent coronary events. Both international and Indian studies have reported low adherence to such therapies. Evidence-based useful interventions include regular physical activity, yoga, intake of healthy diet, smoking and tobacco use cessation and weight management. Pharmacotherapeutic interventions useful are anti-platelet therapy, target oriented lipid lowering therapy with statins, beta blockers and angiotensin converting enzyme inhibitors in patients with impaired left ventricular function. Hypertension and diabetes management with control to targets is important. Novel strategies include use of anticoagulants, anti-inflammatory drugs, and triglyceride lowering for residual risk. Physician and patient level interventions using multifaceted educational, socioeconomic and technological innovations are important to promote life-long adherence to these strategies.

## 1 Introduction

Coronary heart disease (CHD) is most common type of cardiovascular disease and is the leading cause of death worldwide. Mortality due to CHD has decreased in developed countries but India and many other developing countries are still experiencing a significant increase in CHD morbidity and death rates.^1^ Many studies have reported that Indians are more susceptible to coronary artery disease (CAD) and have a higher case-fatality rate than the western populations.^2^

Advances in medical care and prevention have improved survival after the initial event but people with established CHD are at a high risk of subsequent cardiovascular events such as myocardial infarction, stroke, and cardiovascular death. Despite advances in pharmacological treatments and invasive procedures, quality of post-CHD management with better risk factor control and other pharmacological strategies and socioeconomic determinants of health remain independent predictors for fatality in patients with CAD.^3^ Secondary prevention in coronary heart disease (CHD) is prevention of occurrence of recurrent coronary events after clinical diagnosis.^4^ In the present article we shall focus on status of secondary prevention in India and highlight existing and emerging pharmacological therapies that can prevent recurrent coronary events. We also suggest interventions such as digital health and virtual team-based disease management to promote adherence to these lifelong therapies.

## 2 Status of secondary prevention in India

**Fig. 1:**
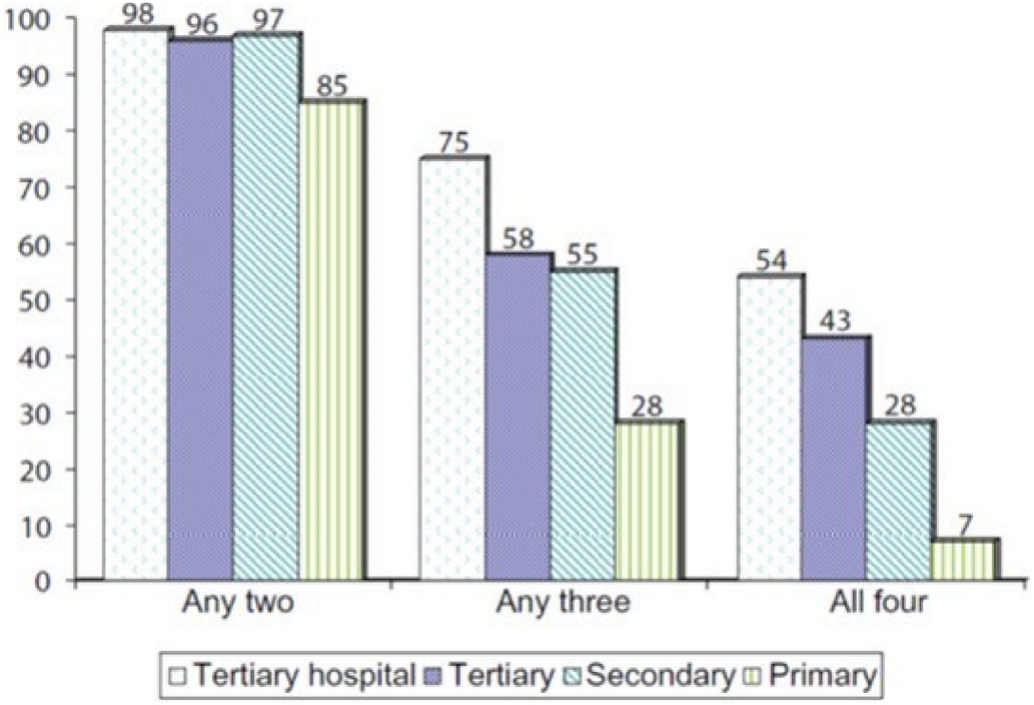
Influence of cardioprotective therapies in reducing 2-year cardiovascular mortality in patients with known CHD.^3^

There is substantial evidence showing that secondary prevention through comprehensive risk factor modification has beneficial effects in patients with CAD, such as a decrease in mortality, reduction in recurrent cardiac events and better quality of life.^5^ It has been suggested that adherence to four cardioprotective medicines: antiplatelet drugs, beta-blockers, angiotensin converting enzyme (ACS) inhibitors and lipid lowering statins can reduce 2 year mortality after acute coronary syndrome from 10% to about 2% (Fig. 1).

Guidelines from American College of Cardiology/American Heart Association (ACC/AHA),^6^^,^^7^ European Society of Cardiology (ESC)^8^ and almost all global CHD management guidelines have recommended aggressive risk factor management with adherence to healthy lifestyle and cardioprotective therapies in all patients who have been diagnosed with acute coronary syndrome (ACS) or have chronic coronary syndromes (CCS).^9^^,^^10^

Status of secondary prevention is poor worldwide and multiple factors, predominantly social determinants of health such as rural location, poverty, illiteracy and low affordability and availability of supportive therapies are important.^3^ In a low-resource setting like India, with poor focus on primary care, secondary prevention of CAD is a difficult task.^4^ Delays in diagnosis, poor quality treatment, and poor adherence to primary and secondary prevention strategies are all symptoms of resource constraints.^11^ The International Council of Cardiovascular Prevention and Rehabilitation (ICCPR) concluded global audit, felt the greatest need for cardiac rehabilitation (CR) exists in India.

The WHO-PREMISE study in 10 countries reported low adherence to drug therapies, particularly ACE inhibitors and statins, in developing compared to the more developed countries.^12^ EUROASPIRE studies were performed in multiple European countries and initial surveys reported low adherence to healthy lifestyles and drug therapies. It was also reported that countries with lower human development index had significantly less adherence to healthy lifestyles (smoking cessation, physical acidity, healthy diet) and secondary preventive cardiac medicines (anti-platelets, beta blockers, ACE inhibitors and statins).^13^ In high-income countries it has been reported that lower socioeconomic status patients have less access to cardiac rehabilitation and lower adherence to healthy lifestyles and secondary prevention drug therapies.^14^

**Fig. 2:**
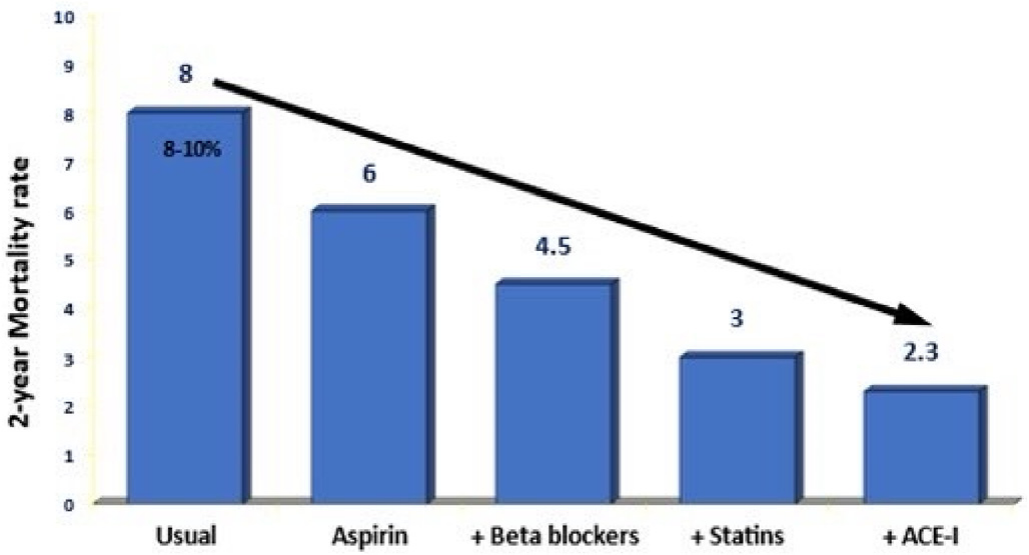
Cardioprotective secondary prevention medicines (aspirin, beta-blockers, statins and ACE inhibitors/ARB) in primary, secondary and tertiary care in Rajasthan.^18^

Prospective Urban Rural epidemiology (PURE) study reported a very low uptake of all the cardioprotective therapies in patients with known IHD and stroke in developing countries compared to more developed countries.^15^ In the South Asian cohort of PURE study reported that low SES patients (low educational status or low wealth index) with IHD or stroke had the lowest consumption of various evidence based therapies at 4–5 years after diagnosis.^16^ A nationwide prescription audit^17^ and a Rajasthan state prescription audit^18^ reported lower secondary prevention therapies in primary care clinics compared to IHD patients in secondary and tertiary care (Fig. 2) A prescription audit among stable IHD patients among a nationally representative sample in China reported that low socioeconomic status was independently associated with lower treatment rates for aspirin, clopidogrel, beta-blockers and statins.^19^

## 3 Secondary prevention interventions

The main focus of secondary prevention is to prevent recurrent coronary events before symptoms appear and to prolong life. It includes disease management combining patient education, multidisciplinary team-based lifestyle management and therapeutic management. Lifestyle management focuses on tobacco (smoking and smokeless) cessation, increased physical activity, weight management and healthy dietary modification while therapeutic management focuses on standard and emerging cardioprotective medical therapies and percutaneous coronary interventions and surgical coronary revascularization.^20^ Class of recommendations and level of evidence of use of various interventions are shown in Table 1.

**Table 1:** Summary of secondary prevention guidelines and class of recommendation.

Lifestyle interventions	Therapeutic agents
• Physical activity (Class I) • Healthy diet (Class I-II) No trans fats (Class I) Reduce saturated fats (Class IIA) Increased MUFA, PUFA (Class IIA) Fruits, vegetables, nuts (Class IB) • Smoking/tobacco cessation (Class I) • Alcohol moderation (Class II) • Weight management (Class II) • Cardiac rehabilitation (Class I)	• Antiplatelets (Class I) o Long term dual (Class IA) vs single (Class 1 B) • Statins (Class I) • Beta-blockers, medium term (Class I) • ACE inhibitors (Class I) or o Angiotensin receptor blockers (Class IIA) • Other drugs classes: nitrates, calcium channel blockers, metabolic modulators, other vasodilators (Class II, III)

### 3.1 Physical activity

Regular physical activity in any form is an important aspect of secondary prevention of CHD because it enhances exercise capacity, ameliorates co-morbid risk factors, and improves quality of life.^21^ Exercise-based cardiac rehabilitation has been shown to lower all-cause and cardiac lethality compared to standard therapies.^21^^,^^22^ All patients should be actively involved in 30–60 min of moderate-intensity physical activity, such as biking or brisk walking. Ancillary physical activity lowers total cholesterol, triglyceride levels, and systolic blood pressure. Exercise-based cardiac rehabilitation programme can be started soon after an acute coronary syndrome or revascularization procedure. Before starting an intensive exercise routine, physicians should assess their patients' cardiovascular status by taking a physical activity history or performing an exercise test. Home based and mobile phone delivered exercise based rehabilitation has emerged to be equivalent to center based cardiac rehabilitation with added benefits of cost reduction and convenience.^23^

### 3.2 Dietary modification: healthy diet

Table 2 shows recommendations from various guidelines for diet. Dietary restrictions vary depending on the type of CAD risk factors. Adults who are at risk for high blood lipids should eat more fruits, leafy greens, whole grains, fish, and low-fat dairy products (healthy diet), while avoiding sugary drinks, sweets, and red meat.^21^ Adults with high blood pressure problem should follow the same dietary restrictions but with low sodium levels. Dietary sodium restriction has also been recommended to reduce the risk of CAD in general population.^24^ Sodium (in the form of salt) causes water retention, and increases sensitivity to blood pressure. Adopting a healthy eating pattern that spreads the daily intake of calories and nutrients over the entire day showed better compliance to the diet. Digital health tools delivered virtually have unique advantages in implementing and monitoring evidence based dietary modification.^25^

**Table 2:** Recommendation for healthy food in various guidelines.

Mediterranean Diet (PREDIMED)	Lancet-EAT Commission Anthropocene diet	American Heart Association	European Society of Cardiology
• Recommended: Olive oil, tree nuts or peanuts, fresh fruits, fatty fish, legumes, white meat, wine • Discouraged: Soda drinks, commercial bakery goods, sweets, pastries, spread fats, red and processed meats	• Diversity of plant-based foods • Low animal source • Balanced intake of unsaturated and saturated fats • Reduced amounts of refined grains, highly processed foods, added sugars	• Greater intake of vegetables, fruits, legumes, nuts, whole grains, fish • Low intake of refined grains, highly processed foods, added sugars	• Recommendations for whole grain products, oily fish, unsalted nuts • High intake of fruits and vegetables, more than 200 g each • Low intake of refined grains, highly processed foods, added sugars

### 3.3 Smoking/tobacco cessation

One of the most cost-effective interventions in secondary prevention is smoking and tobacco-use cessation.^21^ All the guidelines recommend absolute tobacco cessation for CAD secondary prevention. Policy interventions are important and worldwide implementation of WHO Framework Convention for Tobacco Control (FCTC) can lead avoidance of millions of CAD events and can results in saving lakhs of lives.^26^ At the individual level tobacco cessation can be achieved by patient education, nicotine replacement therapies, bupropion, psychotherapy and family support. Avoiding second-hand smoke is equally important. Using daily reminders, rewards for cutting down intake and periodic counselling on symptoms of withdrawal, offered through mobile phone increases success of sustained tobacco cessation.

### 3.4 Alcohol

A controversial issue in secondary prevention relates to alcohol use.^20^ Light to moderate alcohol consumption has been linked to a reduced risk of CAD but the evidence is controversial. It is not recommended by any of the guidelines either for primary or secondary prevention.^4^^,^^15^ A recent cohort study found that alcohol consumption increased the incidence of hypertension by 1.3-fold and CAD by 1.4-fold. Consumption of all amounts of alcohol, including those accepted by current national standards, is associated with increased cardiovascular risk, but there are considerable differences in risk between intake levels.^27^ Building mental resilience and having control on impulsive behaviour reinforces moderation or avoidance of alcohol, specifically binge drinking. Mobile health platforms show promise in behaviour modification for positive change management.

### 3.5 Weight management

Obesity has been associated with an increased risk of CAD death rates, as well as an adverse influence on cardiac function and comorbid lifestyle factors. Maintaining an average weight with a body mass index (BMI) of 23–27 kg/m^2^and a waist circumference of <80 cm for women and <90 cm for men is suggested. The BMI should be measured at each counselling session, according to the American Heart Association (AHA), and then objective reviews and reliable counselling on weight loss programs should be provided. Balance of physical activities and dietary changes is required for long-term weight control, and modest weight loss is associated with changes in cardiac risk factors.^28^ Use of mobile health platforms track daily physical activity duration and intensity. Having the physical instructor call and remind to perform daily tasks increase adherence to weight loss programs. Tracking changes in body parameters with integration of diet, mental wellness and stress management improve exercise tolerance in otherwise sedentary people.

### 3.6 Cardiac rehabilitation

Enrollment in a formal cardiac rehabilitation program is useful to learn the lessons for secondary prevention and better motivation. Studies in India have reported that there is low availability, affordability and awareness of cardiac rehabilitation facilities.^29^ Comprehensive cardiac rehabilitation facility needs physicians, technicians, nurses and other health workers and could be expensive to maintain. Virtual cardiac rehabilitation delivered at home reduces cost, increases referral and uptake. Cochrane review comparing home based to centre based cardiac rehabilitation showed they were equivalent with added benefits to the patient. Studies from the UK and USA have shown that the virtual cardiac rehabilitation is effective.^30^ Disease management through home based digital health tools in heart failure reduced hospital readmission rates and long-term morbidity.^31^ No similar studies exist in India. A low-cost cardiac rehabilitation program has been developed using home-based follow-up following a hospital training protocol.^32^

## 4 Pharmacotherapy

There has been a recent surge in understanding of approaches to long-term management of CAD.^33^ Multiple approaches have been found to be useful and include multiple pillars of intervention. We hereby suggest six pillars of risk mitigation and interventions that focus on LDL cholesterol and residual cholesterol risk, triglyceride risk, thrombotic risk, vascular risk, hyperglycemia and diabetes risk, and inflammatory risk. The targets and therapeutic approaches to achieve targets are shown in Table 3. A combination of these drug-therapies is required to achieve improved outcomes in secondary prevention. Despite the use of all these pharmacological approaches and drugs there remains a substantial residual risk and more studies are required to identify interventions to overcome this risk.

**Table 3:** Reducing cardiovascular risk for secondary prevention.

Biological factors	Biomarker	Intervention/s	Trial evidence	Prevention guidelines
Thrombotic risk	None	Aspirin, P2Y12 inhibitors, rivaroxaban and other novel oral anticoagulants	++++	Yes
Cholesterol and residual cholesterol risk	LDL cholesterol ≥70 mg/dl	LDL cholesterol reduction (ezetimibe, bempedoic acid, PCSK9 inhibition, monoclonal antibodies, small RNA molecules	++++	Yes
Triglyceride risk	Triglyceride ≥150 mg/dl	High dose purified omega-3 fatty acids (icosapent ethyl),	+++	Yes
Lipoprotein(a) risk	Lp(a) ≥50 mg/dl	Monoclonal antibodies- APO(a)-L_RX_, pelacarsen, etc.	–	No
Vascular risk	Blood pressure <130/80 mmHg	Beta blockers, renin-angiotensin system (RAAS) blockers	++++	Yes
Diabetes risk	HbA1c ≥ 6.5%	SGLT-2 inhibitors, GLP-1 agonists	+++	Yes
Inflammatory risk	hsCRP ≥2 mg/dl	Aspirin, colchicine, monoclonal antibodies (e.g., canakinumab)	++	No
LDL low density lipoprotein				

### 4.1 Anti-platelet therapy

Antiplatelet therapy is essential part of medical regimen in acute coronary syndrome as well as for secondary prevention after stabilisation. Multiple randomised trials have clearly shown that dual antiplatelet therapy (DAPT) that includes aspirin and a P2Y_12_ inhibitors should be taken for at least 12 months following acute coronary syndrome. It has also been shown in clinical trials that third generation P2Y_12_ inhibitors (ticagrelor and prasugrel) show additional benefit in terms of decreased ischemic events compared to clopidogrel with an added cost of a slight increase in bleeding, however, the net outcomes are favourable for these drugs. In all the current guidelines, DAPT has typically been recommended for 12 months following acute coronary syndromes with ticagrelor and prasugrel generally preferred over clopidogrel.^34^^,^^35^ The choice of drug as the third generation antiplatelet has been tested in a few trials. The recent MASTER DAPT trial showed that stopping dual antiplatelet therapy after 1 month was noninferior to continuing dual antiplatelet therapy for an additional 2 months in terms of net adverse clinical events and serious adverse cardiac or cerebral events. In addition, abbreviated dual antiplatelet therapy was superior to standard dual antiplatelet therapy in terms of clinically relevant major or minor bleeding.^36^ The ISAR-REACT 5 trial^37^ was a randomised, open-label comparison of prasugrel versus ticagrelor and reported higher rate of the composite primary end point (death, myocardial infarction, or stroke) at 1 year in patients randomised to ticagrelor (9·3 *vs* 6·9%; hazard ratio [HR] 1·36, 95% CI 1·09–1·70) with no significant difference in major bleeding. The 2020 ESC guidelines suggest a Class IIa (level of evidence B) recommendation for prasugrel over ticagrelor in patients with non ST elevation acute coronary syndrome who undergo PCI and are eligible for prasugrel (no prior stroke or transient ischaemic attack).^35^ As for the consideration of single antiplatelet therapy following acute coronary syndrome, the TWILIGHT trial and a metanalysis of 32,000 patients found equivalent risk of MACE with early discontinuation of aspirin within 12 months.^38^^,^^39^ Thus, randomised clinical trials and meta-analyses support dual-antiplatelet therapy following an acute coronary event for at least 12 months and in those with high risk of bleeding, dual antiplatelet therapy for 1 month followed by ticagrelor (or prasugrel) as a single antiplatelet drug can be recommended. Symptom monitoring for potential bleeding complications is possible on digital mobile health platforms. This reduces anxiety, improves prescription confidence and avoids discontinuation of effective antiplatelet therapy.

### 4.2 Anti-coagulation therapy

Approximately 8–10% of patients undergoing for PCI have atrial fibrillation and other indication for an oral anticoagulant.^36^ Several trials have evaluated different strategies and a meta-analysis of these trials reported lower rates of bleeding with a direct oral anticoagulant (DOAC)-based dual antithrombotic therapy than vitamin K agonist triple antithrombotic therapy, but with numerically greater rates of myocardial infarction and stent thrombosis without statistical significance.^36^ Two large randomised trials that compared a DOAC (apixaban or edoxaban) and vitamin K agonist in this setting found lower rates of bleeding with the DOACs.^40^^,^^41^ The 2020 ESC non-ST elevation-ACS guidelines,^42^ recommend 1 week of triple antithrombotic therapy (or until hospital discharge) as a default strategy followed by dual antithrombotic therapy with a DOAC plus P2Y12 inhibitor (typically clopidogrel) until 1 year, at which point DOAC monotherapy can be considered.^29^ Pill reminder short messaging services (SMS) based mobile health platforms improve compliance to anticoagulation therapy. Ensuring adherence reduces risk of stroke from atrial fibrillation. Rivaroxaban, an oral anticoagulant, has traditionally been used to reduce the risk of stroke and systemic embolism in people with nonvalvular atrial fibrillation. The COMPASS trial found that the combination of aspirin with low-dose rivaroxaban (2.5 mg) was effective in preventing major cardiovascular events such as strokes, heart attacks, and death in patients with stable CAD.^43^

### 4.3 Lipid lowering

#### 4.3.1 LDL cholesterol management

Various studies have shown that reducing the level of circulating atherogenic lipoproteins has a major effect on the risk of adverse cardiovascular events. A target LDL cholesterol level lower than 70 mg/dl with an optional target of 55 mg/dl in high-risk secondary prevention has been suggested by European guidelines.^44^ Meta-analyses of multiple statin trials show a dose-dependent relative reduction in cardiovascular events with LDL cholesterol lowering. The Cholesterol Treatment Trialists’ (CTT) Collaborators performed a series of meta-analysis of cholesterol lowering (statins) from 2005 to 2020. Table 4 gives a summary of various statins and recommendations based on target LDL lowering. In the first report which evaluated 14 trials with 90,056 participants there was significant benefit in primary prevention.^45^ In a follow-up study with 18,686 patients with diabetes in these 14 randomized trials, there was a significant 21% proportional reduction in major vascular events per 1 mmol/L (38 mg/dl) reduction in LDL cholesterol in people with diabetes [Odds Ratio (OR) 0.79, 95% confidence intervals (CI) 0.72–0.86] which was similar in those without diabetes (OR 0.79, CI 0.76–0.82) with greater reduction in myocardial infarction and coronary deaths (OR 0.78, CI 0.69–0.87).^46^ In another meta-analyses by CTT collaborators in 2015 among 174,000 participants in 27 randomised trials, lowering of LDL cholesterol by 1 mmol/L reduced major vascular events by 20% (OR 0.80, CI 0.74–0.82).^47^ Wang et al performed a meta-analysis of benefit of lipid lowering in 2020 among 327,037 participants in 27 trials of LDL lowering and reported that reducing LDL cholesterol by 1 mmol/L led to reduced major vascular events by 17% (OR 0.83, CI 0.79–0.88).^48^ Statins are universally recommended for secondary prevention by all the international and national guidelines. High-intensity statin therapy (atorvastatin 40–80 mg/day or rosuvastatin 20–40 mg/dl) must be given to all the patients to achieve targets.^44^^,^^49^^,^^50^ Dietary interventions in lowering lipids when combined with statin therapy, increase compliance and dose dependence.

**Table 4:** Statin therapy (mg/day) for secondary prevention.

Statin	Low intensity <30% LDL lowering	Moderate intensity 30–49% LDL lowering	High intensity >50% LDL lowering
Lovastatin	20 mg	–	–
Pravastatin	10–20 mg	40–80 mg	–
Simvastatin	10 mg	20–40 mg	–
Atorvastatin	–	10–20 mg	40–80 mg
Rosuvastatin	–	5–10 mg	20–40 mg
Pitavastatin	–	1–4 mg	–

If LDL cholesterol remains above 70 mg/dL (55 mg/dl in very high risk patients) despite the use of a maximally tolerated statin, ezetimibe should be added.^44^^,^^49^ Oral bempedoic acid has also emerged as a choice second-line drug in combination with a high-dose statin.^51^ Two monoclonal antibodies against PCSK9, evolocumab and alirocumab reduce LDL cholesterol by 50–70% and have shown major reductions in cardiovascular events in high-risk patients, including within 12 months of acute coronary syndromes.^52^, ^53^, ^54^ Inclisiran, a small molecule based PCSK-9 inhibitor has also shown efficacy in reducing LDL cholesterol and effectiveness in reducing cardiovascular outcomes in CAD.^55^ PCSK-9 inhibition is equally efficient in those with and without diabetes, with a 27% relative risk reduction in cardiovascular death, myocardial infarction, stroke, and hospitalization for unstable angina or revascularization. All these drugs have been approved for use in CAD for LDL cholesterol reduction and outcome benefits. Guidelines recommend their use when LDL cholesterol remains >70 mg/dL despite maximal dose statin plus ezetimibe, or if patients are statin intolerant and ezetimibe alone is ineffective in patients with established CAD.^44^^,^^49^

#### 4.3.2 Triglyceride lowering

Epidemiological studies have reported that raised serum triglycerides is a significant and independent CAD risk factor, but the association is weaker than for hypercholesterolaemia. Meta-analyses suggest that targeting triglycerides may reduce CAD in specific subgroups with high triglycerides and low HDL cholesterol.^56^, ^57^ REDUCE-IT, using high dose purified eicosapantenoic acid-icosapent ethyl, is the first trial to report significant benefits of triglyceride reduction combined with non-triglyceride mechanisms on cardiovascular outcomes.^57^ This drug is now available in India and can be used in secondary prevention and high-risk primary prevention.

#### 4.3.3 Lipoprotein(a) management

Raised lipoprotein(a) [Lp(a)] is now recognized as an important CAD risk factor in epidemiological studies, Mendelian randomized studies and genetic studies.^58^ However, there is no randomized intervention study showing that reducing Lp(a) decreases CAD risk. At present there is no justification for screening the general population for Lp(a), but it may be considered in patients at moderate risk to refine risk evaluation or in subjects with a family history of premature CAD.^33^^,^^44^^,^^58^

#### 4.3.4 Cardiovascular protective agents

Cardioprotective agents work by improving left ventricular ejection fraction, restricting left ventricular hypertrophy, lowering myocardial oxygen demand (which is elevated in patients with CAD due to atherosclerosis) and vascular protection. These drugs include beta blockers, ACE inhibitors/ARBs, calcium channel blockers and nitrates.

#### 4.3.5 Beta blockers

Beta blockers are first-line cardioprotective agents for patients with CAD.^4^ These drugs reduce heart rate, increase diastolic filling time and lower cardiac contractility by restricting β1 and β2 adrenergic receptors. This significant inotropic and chronotropic effect reduces myocardial oxygen demand. Meta-analyses of multiple trials involving more than 24,000 patients who received beta blockers in the convalescent phase of STEMI have shown a 23% reduction in long-term mortality.^6^ When beta blockers are administered early (<6 h) in the acute phase of infarction and continued in the chronic phase of treatment, some of the benefit may result from a reduction in infarct size. Patients with a relative contraindication to beta blockers (e.g., brady arrhythmias, atrioventricular blocks) should undergo a monitored trial of therapy in the hospital. Studies have suggested that beta-blocker therapy be continued for at least 2–3 years after acute coronary event. Long term use is indicated in patients with stable angina, recurrent unstable angina and congestive heart failure.^9^

#### 4.3.6 Inhibitors of renin-angiotensin-aldosterone system (RAAS)

Inhibition of RAAS is essential to prevent cardiac remodelling in post-acute coronary syndromes (STEMI or NSTEMI) patients. Treatment with ACE inhibitors such as captopril, enalapril, ramipril, trandolapril, or zofenopril for 1 month to 1 year in acute myocardial infarction improves left ventricular ejection fraction.^59^ Findings from the HOPE trial showed that treatment with ramipril 10 mg/day for 5 years reduces the incidence of death, myocardial infarction, cardiac arrest, and heart failure in patients with CAD, stroke, peripheral vascular disease, or diabetes without evidence of ejection fraction or heart failure.^60^ The results of the EUROPA trial showed that treatment with perindopril at 8 mg/day for 4.2 years reduced cardiovascular events among patients with stable coronary heart disease.^61^ Based on the results of these studies, ACE inhibitor (ramipril, perindopril) for all STEMI patients with ejection fraction <40%, renal dysfunction, or diabetes regardless of ejection fraction should be given if no contraindication exist.^42^ The VALIANT trial results suggest that valsartan may be used as an alternative to an ACE inhibitor (in ACE inhibitor intolerant patients) for the long-term management of patients with left ventricular dysfunction after STEMI.^62^ The STEP study demonstrated that intensive BP treatment (systolic BP target, 110 mmHg to <130 mmHg) benefits older hypertensive patients (60–80 years) and reduces the risk of cardiovascular events than standard treatment.^63^ A recent meta-analysis of six randomised clinical trials including 27,400 hypertensive patients (aged ≥60) found that intensive systolic blood pressure lowering (systolic BP < 140 mm Hg) reduced the incidence of major adverse cardiovascular events by 21%.^64^

### 4.4 Diabetes management

Proper management of type 2 diabetes is crucial to prevent complications in CAD secondary prevention. Two classes of drugs, SGLT2 inhibitors and GLP1 receptor agonists, have shown long term benefit in secondary prevention.^65^ Results of meta-analyses of randomized controlled trials suggest that while GLP1 receptor agonists are more useful for primary prevention, SGLT2 inhibitors are better in secondary prevention, especially in patients with impaired left ventricular function.^66^^,^^67^ Braunwald concluded that SGLT2 inhibitors are responsible for paradigm shifts in care of patients at high risk of heart failure-CAD secondary prevention, or having heart failure.^68^ SGLT2 inhibition improves cardiovascular outcomes in patients with heart failure over a wide range of ejection fractions, regardless of whether the patients have type 2 diabetes. These drugs are also recommended as part of secondary as well as CAD primary prevention therapies.

### 4.5 Anti-inflammatory agents

Inflammation after acute coronary syndrome significantly contributes to recurrent ischemic events. Lipid lowering with rosuvastatin was effective in primary prevention with raised hsCRP levels (indicative of underlying inflammation).^69^ Secondary prevention trials with a novel monoclonal antibody targeting interleukin-1b (canakinumab) reported significant benefit in secondary prevention.^70^ Anti-inflammatory treatment with colchicine after acute coronary syndrome has recently shown some promise. In the COLCOT trial colchicine showed 23% reduction of MACE compared to placebo at 30 days in MI patients.^71^ Similar findings were observed among patients with chronic coronary syndrome in the LoDoCo2 trial.^72^ At present there is no recommendation for colchicine in CAD secondary prevention.

## 5 Adherence to secondary prevention

One of the most important aspects of secondary prevention is adherence to therapies. Adherence is defined as remaining attached to the medication regime and adequate adherence is use of therapies >80% of times.^73^ Studies have reported that in chronic diseases only 50% patients are adherent to therapies at 12 months and just 20% take medications in appropriate dose.^74^ Observational studies from all over the world, especially developing countries, have shown that most patients either not prescribed the full spectrum of lifestyle related interventions or pharmacological therapies.^19^ Studies in US and North America have reported that less than 50% patients adhere to appropriate therapies at five-years post ACS while the rates are much lower (<10%) in developing and underdeveloped countries of Asia, Africa and Latin America.^15^, ^75^ There are numerous barriers to adherence and secondary prevention (Table 5). Although universally important, these factors are more relevant in middle- and low-income countries such as India.^3^

**Table 5:** Barriers to adherence and secondary prevention.

Community level barriers	Health system barriers	Provider barriers	Patient related factors
• Low perceived needs • Lack of heart-friendly infrastructure • Government policies for food, tobacco • Media apathy	• Resource constraints • Poor access and availability • Lack of advocacy	• Lack of understanding pf patient needs • Prescribing complex regimens • Failure to explain benefits and side effects • Lack of focus on lifestyle changes • Lack of continuity of care • Low clinical referrals • Clinician perceptions • Over treatment	• Older age • Female gender • Low socioeconomic status • Social isolation • Co morbidities • Multiple stakeholders • Disparate messages • Finance and insurance • Geographic factors

### 5.1 Interventions to promote adherence

Outcomes of interventions to promote adherence are limited. A number of interventions directed at health care system, healthcare professionals and patients have been suggested (Table 6). There is some evidence that technology-based interventions, pharmacists’ level interventions and health-worker based interventions are useful in promoting adherence and influence intermediate outcomes.^76^

**Table 6:** Interventions to promote adherence.

System level	Provider level	Individual level
• Prioritization of secondary prevention • Education of providers and patients • Simplify referral and enrolment processes • Increase resources for secondary prevention services • Increase capacity and capabilities of health care providers • Increase capacity of programs	• Improve education • Teach adherence promotion techniques • Motivation • Cost awareness • Single pill combinations, polypills	• Discuss advantages/disadvantages of drugs • Motivational interviewing • Patient choice • Recommend intake in written format • Reminders using nurses, pharmacists and family members • Discuss adherence at each visit • Continuous counselling strategies, telephone etc. • Small number of single daily doses • Fixed combinations • Dose-dispensed medicines • Door-step level care (non-physician health workers, community health workers, technology)

An important factor that promotes adherence in CHD primary and secondary prevention is self-management of risk factors.^21^ Adherence to healthy lifestyles and pharmacological therapy in asymptomatic high-risk individuals is a herculean task and patient empowerment and personalized medicine to support lifelong adherence to lifestyle changes and drug therapies can be useful. Technology-based strategies to promote adherence to healthy lifestyles and drug therapy are available and given the universality of smart-phone devices the potential for this personalized approach is enormous.

## 6 Digital health tools in secondary prevention

The digital health system is currently a thriving health care system, primarily for the management of chronic diseases, particularly after the COVID-19 pandemic. Digital healthcare systems can potentially address the failures of traditional treatments and therapies by efficiently sharing information with patients, providers, and decision makers, overcoming the barrier of geographic location to expand access to health care, delivering personalized care more precisely; reducing travel and treatment costs, improving compliance and patient adherence; and enabling remote monitoring of patients.^77^ Digital health systems are divided into several categories according to the objectives and criteria, including telehealth and telemedicine, medical education, digital tools for diagnosis or decision making, devices to regulate or improve physiological functions, health informatics, and so on.^78^ From the patient's perspective, digital health technologies offer valuable resources to develop self-management skills using diverse online platforms, mobile, social media, and wearable devices.^79^ Despite the potential of healthcare delivery, the implementation and sustainability of digital healthcare interventions is very difficult to create a universal “digital prescription” for the management of chronic diseases due to the variable socio-cultural and economic status of people and the different laws and policies of different countries to adopt digital health care interventions.^80^ Besides, digital health interventions are very complex and their practical implementation is very challenging due to various factors such as reliability/technical stability of electronic sensors and data transmissions; transparency of algorithms for autonomous decisions; access and usability; reorganization of workflow/infrastructure; poor implementation planning and security threats in data transmission and storage.^81^ Because of these barriers, only a limited number of successful, evidence-based digital health interventions are beyond the pilot or feasibility stage (Table 7). The efficacy of these digital health applications was assessed using different disease control outcome measures, such as disease symptoms, adverse events, exercise capacity, disease-related mortality, self-efficacy, quality of life assessment, economic outcomes and treatment adherence.^82^ Large efforts have also been made toward the overall goal of leveraging data to improve health information exchange and care coordination, but unfortunately, these efforts are significantly fragmented and provider-centric rather than patient-centric.

**Table 7:** Outcomes of various digital health interventions in chronic disease management.

Sl No	Title of the study	Type of study	Intervention studied	Main outcomes
1.	Effect of mobile applications on blood pressure control and their development in China: a systematic review and meta-analysis.^83^	Systematic review/meta-analysis	Mobile application for blood pressure control	The results of all 18 trials showed a significant additional decrease in both SBP (mean difference [MD] = 8.12 mmHg, 95% confidence interval [CI]: 11.47 to 4.77 mmHg; P < 0.001 and DBP (MD = 6.67 mmHg, 95% CI: 8.92 to 4.41 mmHg; P < 0.001.
2.	Effectiveness of technology-assisted cardiac rehabilitation: A systematic review and meta-analysis.^84^	Systematic review/meta-analysis	Technology-assisted interventions on modifiable coronary risk factors, exercise capacity, quality of life, psychological outcomes and adherence	The study showed equivalent outcomes to centre-based CR in the modifiable coronary risk factors such as systolic and diastolic blood pressure, total cholesterol, waist circumference, and BMI. While the technology-assisted interventions did not improve anxiety and depression, they did improve exercise capacity (peak VO2 and 6MWT). The quality of life and psychological outcomes of the technology-assisted interventions were not superior to centre-based CR. The adherence to exercise training was similar to that of the centre-based CR.
3.	Effectiveness of workplace interventions to reduce the risk for type 2 diabetes: A systematic review and meta-analysis.^85^	Systematic review/meta-analysis	Workplace type 2 diabetes (T2D) prevention programs (DPP)	This study included a total of five randomised controlled trials. DPP-based interventions were 3.85 times more likely to result in a weight loss of more than 5% (4 RCTs; risk ratio [RR] = 3.85; 95% CI, 1.58 to 9.38; p < 0.05), and 9.36 times more likely to result in a weight loss of 7% (2 RCTs; RR = 9.36; 95% CI, 2.31 to 37.97; p < 0.05).
4.	Impact of technology-based patient education on modifiable cardiovascular risk factors of people with coronary heart disease: A systematic review.^86^	Systematic review	Technology-based patient education on modifiable cardiovascular risk factors	This study included eighteen quantitative studies. All of the interventions improved modifiable cardiovascular risk factors in the following ways: a) Telephone follow-up was found to have a significant positive effect on physical activity, diet, and weight, as well as lowering CHD risk scores. b) Text messaging increased physical activity, vegetable and fruit consumption, weight, LDL, triglycerides, haemoglobin A1c, and CHD risk scores significantly. c) Websites had a significant positive effect on physical activity and diet in people with CHD. d) In people with CHD, smartphone applications had a significant positive effect on blood pressure and triglycerides.
5.	Online patient education interventions in type 2 diabetes or cardiovascular disease: A systematic review of systematic reviews.^87^	Systematic review of systematic reviews	Online patient education in Type 2 DM and CVD	This study included 23 systematic reviews of biological, behavioural, psychological, knowledge, and self-efficacy measurements. Despite a mixed overall picture, all of these indicators for individuals with Type 2 DM and CVD have demonstrated benefits from online patient education. Regardless of effect size, the study's outcomes consistently indicated improvements with the intervention in terms of weight, physical activity, knowledge, social support, and quality of life.
6.	Social media interventions targeting exercise and diet behaviours in people with noncommunicable diseases (NCDs): A systematic review.^88^	Systematic review	Social media interventions targeting physical activity and/or diet for people with NCDs	This review included eight studies, including five RCTs and three pilot or feasibility studies involving people with severe mental illness, cancer, CVD, COPD, and type 2 diabetes. Exercise behaviours (such as step count and exercise capacity) were significantly improved in four out of five RCTs. However, dietary behaviour was only evaluated in two studies with non-significant outcomes.
7.	Strategies to promote the use of online health applications for early detection and raising awareness of chronic diseases among members of the general public: A systematic literature review.^89^	Systematic review	Online health applications for early detection and raising awareness of chronic diseases	The review included 47 studies, 32 of which used a strategy to promote the use of their online intervention, and 27 of which reported strategies to keep users engaged. Various engagement strategies, particularly online advertisements and reminder messages, were found to improve user engagement, exposure to online health interventions, and the overall impact of such interventions. There was no formal evaluation of the effectiveness of the engagement strategies in any of the studies.
8.	The effectiveness of electronic health interventions on blood pressure control, self-care behavioural outcomes and psychosocial well-being in patients with hypertension: A systematic review and meta-analysis.^90^	Systematic review/meta-analysis	Electronic health interventions on blood pressure control, self-care behavioural outcomes and psychosocial well-being	In all, 14 papers were incorporated into this study. In thirteen trials, eHealth interventions resulted in a significant decrease in both systolic and diastolic blood pressure (MD: −5.96 mmHg, 95% CI: −9.21 to −2.70, p.001) as well as heart rate (MD: −3.35 mmHg, 95% CI: −6.36 to −0.35, p.05). The proportion of patients with insufficient blood pressure control was significantly reduced by eHealth interventions (RR: 0.69, 95% CI: 0.57–0.84, p.001), as was the patients' body weight (MD: −1.08 kg, 95% CI: −2.04 to −0.13, p.05). eHealth programmes effectively decreased sodium intake in terms of self-care behavioural outcomes.
9.	What makes digital interventions effective for exercise promotion: A systematic review of reviews and meta-analysis.^91^	Systematic review of reviews and meta-analysis	Digital health interventions for prevention and management of CVD	This study included 29 RCTs that promoted exercise or cardiovascular fitness. The study's findings revealed a significant improvement in exercise and cardiovascular fitness when compared to the control group (MD = 0.44, 95% CI [0.16, 0.64]. Subgroup analyses revealed significant impacts when interventions included at least four therapeutic strategies (i.e., patient education, self-monitoring, intervention tailoring, and use of additional services) (MD = 0.67, 95% CI [0.27, 1.08]) and when they specified an organised theoretical framework or clinical model to guide their intervention (MD = 0.43, 95% CI [0.25, 0.62]).

## 7 Conclusions

Secondary prevention in coronary heart disease (CHD) is crucial to reduce mortality and morbidity from this condition. High level of adherence to secondary prevention interventions including aggressive lifestyle changes and appropriate pharmacotherapy can lead to a significant decline in recurrent coronary events. Suggested interventions include regular physical activity, intake of healthy diet, smoking and tobacco use cessation, weight management and alcohol moderation. Pharmacotherapy interventions found useful are anti-platelet therapy (short-term dual and long-term single), target oriented lipid lowering therapy with statins (ezetimibe, bempedoic acid or PCSK9 inhibitors in statin non-responsive or intolerant), beta blockers (medium to long term) and angiotensin converting enzyme (ACE) inhibitors (angiotensin receptor blockers in ACE inhibitor intolerant). Novel strategies include use of anticoagulant and anti-inflammatory drugs, and lowering triglycerides and lipoprotein(a). Physician and patient level interventions are important to promote life-long adherence to these strategies. Digital health interventions delivered through adoption of technology and specialized follow ups by multidisciplinary teams have evidence for preventing hospital re-hospitalization and potentially reduce mortality in the long run. Despite the fact that the use of digital technologies is still in its early stages, continued efforts in this field may result in the introduction of evidence-based digital health applications for chronic disease management, which could be major disruptors to healthcare markets in the coming years.

## Author declaration

a) We the undersigned declare that this manuscript is original, has not been published before and is not currently being considered for publication elsewhere.
b) We wish to confirm that there are no known conflicts of interest associated with this publication and there has been no significant financial support for this work that could have influenced its outcome.
c) We confirm that the manuscript has been read and approved by all named authors and that there are no other persons who satisfied the criteria for authorship but are not listed. We further confirm that the order of authors listed in the manuscript has been approved by all of us.
d) We agree to transfer copyright to the Indian Heart Journal upon the acceptance of the manuscript for publication.
e) We understand that the Corresponding Author is the sole contact for the Editorial process (including Editorial Manager and direct communications with the office). He is responsible for communicating with the other authors about progress, submissions of revisions and final approval of proofs.

## Declaration of competing interest

The authors declare that they have no known competing financial interests or personal relationships that could have appeared to influence the work reported in this paper.
